# Designing and optimizing an intelligent health informatics management platform for patients undergoing chemotherapy for breast cancer: from needs assessment to pilot testing

**DOI:** 10.3389/fonc.2026.1865670

**Published:** 2026-10-22

**Authors:** Xiaosong Yu, Lin Zhang, Li Yang, Shaolin Chen, Xiaohua Wang

**Affiliations:** 1 Department of Nursing, Affiliated Hospital of Zunyi Medical University, Zunyi, China; 2 School of Nursing, Zunyi Medical University, Zunyi, China; 3 Information Department, Affiliated Hospital of Zunyi Medical University, Zunyi, China; 4 School of Medical Information Engineering, Zunyi Medical University, Zunyi, China

**Keywords:** breast cancer, chemotherapy-induced myelosuppression, health informatics management platform, mHealth, remote monitoring

## Abstract

**Objective:**

To design and optimize a health informatics management platform embedded in an official WeChat public account—a widely used social media and messaging application in China—to support chemotherapy-induced myelosuppression (CIM) management and patient self-management among breast cancer patients undergoing chemotherapy.

**Methods:**

Guided by a user-centered design approach, the platform was developed and refined in three stages: needs assessment, expert review and iterative refinement, and pilot feasibility testing.

**Results:**

Most patients (88.6%) and clinicians (93.9%) recognized the need for such a platform. In the pilot evaluation, the mean overall MAUQ score was 3.63 ± 0.32, suggesting moderate usability and room for further refinement. During pilot testing, the alert system generated 11 CIM-related alerts and delivered corresponding risk-stratified guidance.

**Conclusions:**

The platform may support personalized CIM management by enabling remote monitoring, automated alerts, and risk-stratified guidance. Further studies with larger samples and longer follow-up are needed to evaluate its effectiveness in improving patient self-management and clinical follow-up efficiency.

## 1 Introduction

Breast cancer remains the most commonly diagnosed malignancy among women worldwide and a leading cause of cancer-related death in this population (1).

According to GLOBOCAN 2022 estimates from the International Agency for Research on Cancer (IARC), breast cancer accounted for approximately 2.30 million new cases globally in 2022, representing 11.5% of all newly diagnosed cancers, and caused 666,103 deaths, accounting for 6.8% of all cancer-related deaths (2). The global incidence of breast cancer continues to increase, placing a growing burden on public health systems, with projections suggesting that more than 3 million new cases and over 1 million deaths will occur annually by 2040 (3, 4).

Chemotherapy remains a cornerstone of breast cancer treatment and is widely used in neoadjuvant, adjuvant, and metastatic settings (5–8). However, chemotherapy-induced myelosuppression (CIM) is a frequent and clinically important adverse event that can compromise treatment delivery, therapeutic efficacy, and quality of life. It also increases the risk of infection, anemia, and bleeding, thereby imposing substantial physical and psychological burdens on patients (9–13).

Among patients with breast cancer receiving chemotherapy, the cumulative incidence of CIM has been reported to be as high as 80%-90% (9, 13, 14). Timely, systematic, and standardized management can reduce the risk of complications, enhance chemotherapy safety, and support treatment adherence (15, 16). However, CIM management in routine practice still relies largely on hospital-based follow-up and passive patient reporting (9, 17), resulting in several challenges, including infrequent monitoring, poor adherence, limited individualization, and inefficient use of healthcare resources. These limitations may delay the recognition of clinical deterioration, postpone interventions, and ultimately contribute to suboptimal outcomes (9, 18, 19).

In recent years, mobile health (mHealth) technologies have been increasingly adopted in patient education, nursing care, disease prevention, and treatment support (20–22). In chronic disease and cancer care, mHealth interventions have shown potential to deliver individualized support, facilitate early warnings of disease progression or adverse events, improve adherence, reduce acute complications, and promote long-term health management. They may also provide more accessible and cost-efficient care options for both patients and healthcare providers (23–25).

Despite this potential, mHealth tools continue to face challenges in sustaining user engagement, enabling intelligent monitoring, and delivering personalized support (23), particularly in low- and middle-income countries, where linguistic, health-system, and technological barriers may hinder implementation (26, 27). Within breast cancer care, recent mHealth initiatives have addressed different points along the care continuum. A published pilot randomized trial used a smart pill bottle and text messaging to support capecitabine adherence and patient-reported symptom monitoring (28), whereas an ongoing mHealth-enabled patient-navigation trial is evaluating strategies to improve breast cancer screening and early detection (29). Although these approaches broaden digital support across treatment and screening, they do not specifically address CIM-related hematologic monitoring and management.

To address this unmet need, this study used a user-centered design (UCD) approach to develop the Breast Cancer Chemotherapy-Induced Myelosuppression Management Platform (BCI-MP). Unlike the aforementioned applications, the BCI-MP integrates reminders for complete blood count testing and result reporting with algorithm-based CIM grading, automated alerts, and risk-stratified guidance, thereby linking objective laboratory data with remote monitoring and clinical decision support during chemotherapy intervals.

## 2 Materials and methods

This study was conducted through multidisciplinary collaboration to design and preliminarily evaluate a health informatics management platform for chemotherapy-induced myelosuppression (CIM) in patients with breast cancer. A user-centered and iterative development framework was used, comprising three sequential phases: (1) a needs assessment among patients and healthcare professionals to identify functional requirements; (2) expert panel consultations to refine the core modules and interaction logic; and (3) pilot testing to assess technical functionality and initial usability in clinical practice. This structured process was designed to align the platform functions with clinical workflow requirements and user experience, thereby informing subsequent implementation and optimization.

This study was approved by the Ethics Committee of the Affiliated Hospital of Zunyi Medical University (approval No. KLLY-2023-160; approved on December 31, 2023). Written informed consent was obtained from all participants. The study was registered in the Chinese National Medical Research Registration and Information System (ChiCTR2400085514).

### 2.1 Stage 1: needs assessment and functional planning

#### 2.1.1 Needs assessment

Two tailored needs assessment questionnaires were developed: one for patients with breast cancer undergoing chemotherapy and another for healthcare professionals working in breast surgery departments. Each questionnaire collected demographic information, previous experience with myelosuppression, prior use of digital health tools, willingness to use the platform, and perceived functional needs. Functional need items were rated on a 5-point Likert scale. Although the questionnaires were designed primarily to capture perceived needs rather than formal psychometric constructs, both showed good internal consistency in this study population (Cronbach’s α = 0.948 for healthcare professionals and 0.759 for patients). Detailed information on questionnaire structure and scoring is provided in **Supplementary Material 1**.

A cross-sectional needs assessment was conducted using purposive sampling. Healthcare professionals working in breast surgery departments and patients with breast cancer during chemotherapy intervals were recruited. Two instruments were administered to each participant: a general information form and a group-specific needs assessment questionnaire. Eligible healthcare professionals were currently practicing in breast surgery departments and provided voluntary informed consent; trainees and rotating staff were excluded. The patient inclusion criteria were: (1) age ≥18 years; (2) pathologically confirmed breast cancer (TNM stage I–IV); (3) receipt of chemotherapy within the previous 12 months, irrespective of the specific chemotherapy regimen; and (4) willingness to participate and provision of written informed consent. No additional patient-level exclusion criteria were applied. Eligibility was not based on anticipated regimen-related myelosuppression risk, and independent smartphone use was not required because the needs assessment was available in both online and paper formats. Questionnaires with implausibly short completion times, patterned responses, or missing data were excluded after independent review by two researchers.

To broaden participant coverage and reduce access-related barriers, data were collected using a combined online and offline approach (30). Online questionnaires were distributed through professional and patient communication groups, whereas paper-based questionnaires were administered in clinical settings to accommodate participants who were less familiar with digital tools. All responses were independently reviewed by two researchers, and duplicate or inconsistent entries were removed to enhance data integrity and reliability.

#### 2.1.2 Preliminary functional planning

Based on the needs assessment results, the research team analyzed the questionnaire data to identify priority functions and content areas. These findings informed the preliminary intervention process (**Figure 1**), the initial functional module framework of the Breast Cancer Chemotherapy-Induced Myelosuppression Management Platform (BCI-MP), and the prototype of the intelligent interaction engine. The initial design incorporated key functions, including blood test reminders, automated alerts, health education, and clinician-patient communication, into a structured platform framework. Highly rated functional items were prioritized in the initial interface design to facilitate subsequent expert review. The initial functional modules and intelligent interaction engine are presented in **Supplementary Tables S1, S2** in **Supplementary Material 2**.

**Figure 1:**
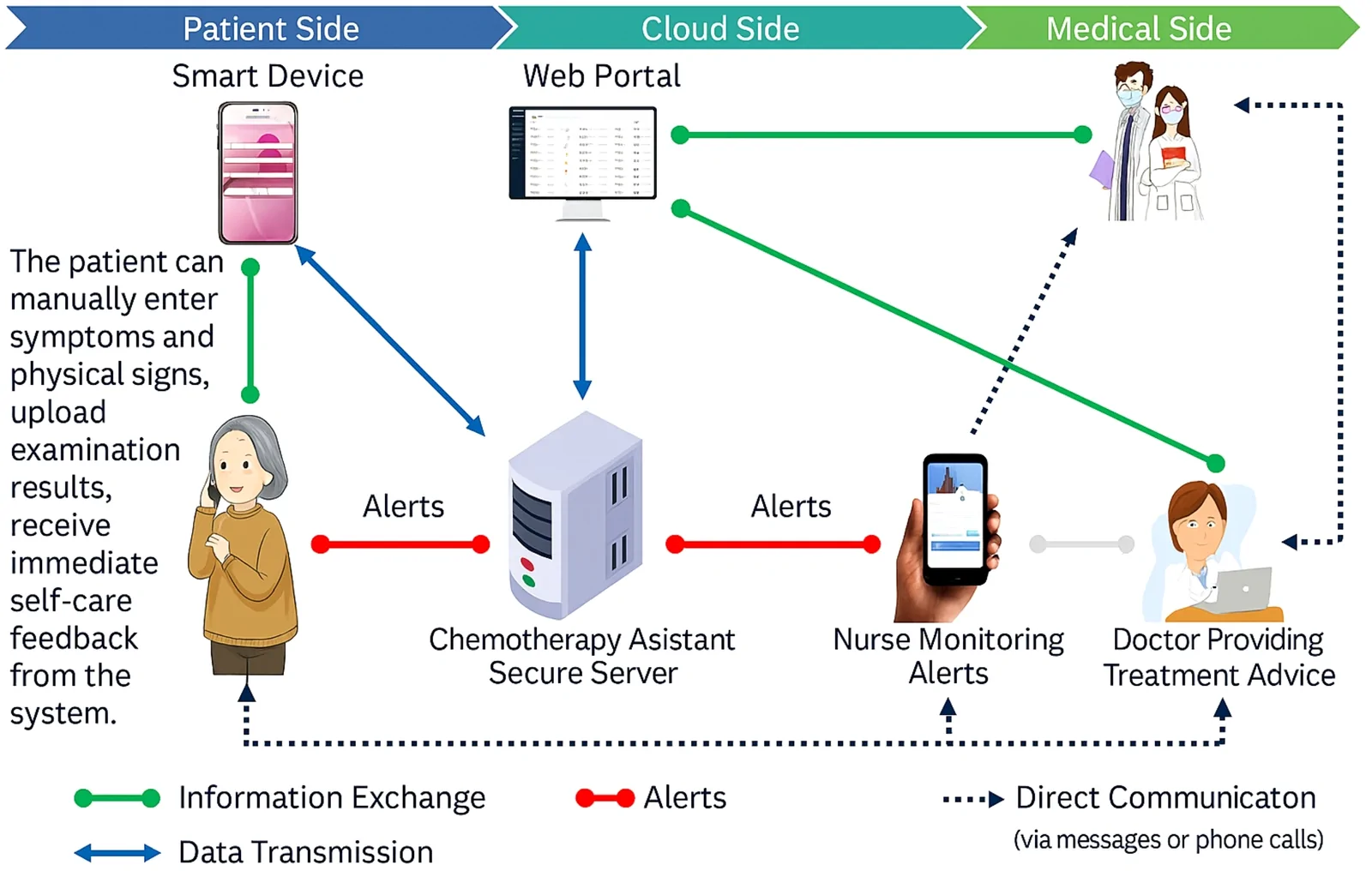
Workflow of the BCI-MP intervention. It illustrates the data flow and interaction among patients, the cloud-based BCI-MP platform, and healthcare providers during the intervention process.

### 2.2 Stage 2: expert panel meetings

Two rounds of structured expert panel meetings were convened to strengthen the methodological rigor and practical relevance of the platform design (31). Thirteen multidisciplinary experts from breast surgery, nursing, medical informatics, psychology, and nutrition reviewed and refined the draft functional modules. The expert authority coefficient (Cr) was calculated as Cr = (C_a + C_s)/2, where C_a represents the basis for judgment and C_s represents the degree of familiarity with the content. Cr values ranged from 0.75 to 1.00, with a mean of 0.88, indicating a high level of expert authority. Each meeting was chaired by one investigator and documented by another. Based on expert feedback, the research team iteratively refined and finalized the BCI-MP module framework. Expert characteristics are summarized in **Table 1**.

**Table 1:** Characteristics of the expert panel (n = 13).

Category	Item	n	%
Specialty	Breast and Thyroid Surgeon	5	38.4
	Breast and Thyroid Nurse	4	30.8
	Medical Informatics	2	15.4
	Psychiatry	1	7.7
	Department of Nutrition	1	7.7
Years of Work Experience	5–14 years	6	46.1
	15–24 years	3	23.1
	≥25 years	4	30.8
Educational Background	Doctorate	5	38.4
	Master’s Degree	4	30.8
	Bachelor’s degree	4	30.8
Professional Title	Senior	7	53.9
	Intermediate	6	46.1

### 2.3 Stage 3: platform development and pilot operation

#### 2.3.1 Platform development

The chemotherapy-interval myelosuppression management platform developed in this study was embedded in the department’s official WeChat public account and implemented as the primary system interface.

The platform was initially developed in a Windows 10 environment using Apache 2.4, MySQL 8, and PHP 7.2. It was subsequently deployed on a CentOS 8 server with Nginx 1.24.0 and PHP 7.3 to improve system stability and compatibility. Back-end functions were developed in PHP, whereas the front end was built using HTML, CSS, and JavaScript to support dynamic user interaction. SQL was used for data queries and database operations. The system adopted a front-end/back-end separation architecture to enhance modularity, maintainability, and scalability. This architecture supported stable platform operation and provided a technical basis for subsequent optimization and iteration.

#### 2.3.2 Pilot operation

From February 1 to March 8, 2024, a pilot test was conducted in the Breast Surgery Department of a tertiary Grade A hospital in Guizhou Province. The pilot test aimed to verify the technical functionality of the platform and assess the alignment between platform functions and user needs. Eight patients with breast cancer undergoing their first cycle of chemotherapy were recruited through convenience sampling to use the platform and provide feedback on their experience.

Platform usability was evaluated using the validated Chinese version of the mHealth App Usability Questionnaire (MAUQ) developed by Zhao et al. (32). The MAUQ consists of 21 items across three domains: system ease of use and satisfaction, information architecture, and perceived usefulness. Each item was rated on a 7-point Likert scale. In this study, lower scores indicated higher usability. After one month of use, the questionnaire showed high internal consistency in the pilot sample (Cronbach’s α = 0.912). Details of the scale items are provided in **Supplementary Table S3** in **Supplementary Material 2**.

Operational metrics were extracted from system server logs, including the number of blood test result uploads, views of health education materials, auto-triggered alerts, and SMS reminders sent. These data were used to assess usage frequency and system responsiveness. All data were collected by trained researchers and independently double-verified for accuracy and completeness. Quantitative data were analyzed using SPSS version 29.0 and are presented as mean ± standard deviation (mean ± SD) or frequency and percentage (n, %).

## 3 Results

### 3.1 Needs assessment

#### 3.1.1 Patients

A total of 346 questionnaires were distributed to patients, and 306 valid responses were obtained, yielding a valid response rate of 88.44%. Patients ranged in age from 25 to 80 years, with a mean age of 50.62 ± 9.29 years. More than half of the patients were aged 50 years or older (161/306, 52.61%). All participants were women, and most had a junior high school education or below (256/306, 83.66%). Additional demographic and clinical characteristics of the patients are summarized in **Supplementary Table S4** in **Supplementary Material 2**.

Patients reported substantial needs for functions related to disease-specific knowledge, complete blood count monitoring reminders, personalized health guidance, and clinician-patient communication. Among the respondents, 271 (88.56%) indicated a need for the BCI-MP platform, and 252 (82.35%) expressed willingness to use it. Detailed scores for each functional domain are presented in **Table 2**.

**Table 2:** Specific demand scores of patients for BCI-MP (N = 306).

No.	Item	Mean ± SD
1	I need to access disease-related knowledge and coping strategies at any time.	4.85 ± 0.42
2	I need to know whether my complete blood count results are normal.	4.83 ± 0.50
3	I need to receive reminders before each follow-up complete blood count test.	4.82 ± 0.53
4	If my symptoms are severe, I need a quick way to contact my doctor and return to the hospital for treatment.	4.79 ± 0.62
5	I need the platform to provide me with targeted advice and guidance based on abnormal complete blood count results.	4.76 ± 0.77
6	I need to see the results of each of my blood routine tests after the start of chemotherapy.	4.57 ± 0.74
7	I need to maintain contact and communication with medical staff.	4.31 ± 0.93
8	I need to maintain contact and communication with fellow patients.	4.24 ± 0.86

Items were rated on a 5-point Likert scale (1–5), with higher scores indicating greater perceived need.

#### 3.1.2 Healthcare professionals

A total of 180 questionnaires were distributed to healthcare professionals, of which 164 were valid, yielding a valid response rate of 91.11%. Respondents ranged in age from 23 to 59 years, with a mean age of 29.91 ± 5.36 years, and had 1 to 34 years of work experience, with a mean duration of 10.62 ± 6.42 years. Most respondents held a bachelor’s degree or higher (135/164, 82.31%). Additional demographic and professional characteristics of the healthcare professionals are summarized in **Supplementary Table S5** in **Supplementary Material 2**.

Healthcare professionals reported substantial needs for functions such as patient monitoring, graded intervention, visualization of blood test data, and clinician-patient communication. Among the 164 respondents, 154 (93.90%) considered the BCI-MP platform necessary, and 140 (85.37%) expressed willingness to use it in clinical practice. Physicians reported greater willingness to use the platform than nurses. Detailed scores for each item are presented in **Table 3**.

**Table 3:** Specific scores of medical staff’s needs for BCI-MP (N = 164).

No.	Item	Mean ± SD
1	I need to promptly contact patients who have developed severe myelosuppression and arrange for their return to the hospital for treatment.	4.83 ± 0.85
2	I need to remind patients to review their complete blood count at the appropriate time points.	4.81 ± 0.77
3	I need to provide patients with disease-related knowledge and coping strategies.	4.78 ± 0.75
4	I need the platform to visualize patients’ longitudinal complete blood count data.	4.76 ± 0.79
5	I need to provide targeted guidance for patients with different grades of myelosuppression.	4.68 ± 0.82
6	I need the platform to automatically grade and alert for patients who have developed myelosuppression.	4.57 ± 0.83
7	I need to maintain contact and communication with patients	4.57 ± 0.81
8	I need to promptly review the results of patients’ myelosuppression status	4.34 ± 1.03

Items were rated on a 5-point Likert scale (1–5), with higher scores indicating greater perceived need.

### 3.2 Expert meetings

#### 3.2.1 First round

The first expert panel meeting focused on the determination of BCI-MP functional modules and content. Experts provided concrete and actionable recommendations for optimization, particularly regarding information completeness, interaction logic, and personalization. After the meeting, the research team systematically reviewed and synthesized all feedback. Expert recommendations were integrated with internal team discussions to revise the initial platform draft. Detailed expert feedback and corresponding revisions are summarized in **Table 4**.

**Table 4:** Expert opinions and handling results from the first expert meeting.

Function and content items	Expert opinions	Feedback handling
1–4 Member Management	Expert 5: Some patients may enter incorrect information or change their treatment information midway. The medical staff interface should provide an option to modify this information.	Accepted. An edit function (1-4-21) has been added to the medical staff backend, allowing modification of patient disease information.
1–5 Health Education	Expert 7: Provide video and audio upload capabilities, so that videos of “cancer-fighting heroes” can be uploaded to boost confidence in overcoming the disease.	Not adopted. The existing editing function already supports video and audio uploads; therefore, no additional modification was considered necessary.
1–8 Bone Marrow Suppression Grading Settings Handling	Expert 3: After grading according to the severity of symptoms, it is not only severe patients who require intervention; patients with milder symptoms also need early intervention to prevent disease progression. It is recommended to have an automated prompt for each level. Expert 11: In the intelligent alarm system, threshold values can be set for each level, and each level can have different corresponding suggestions and recommendations.	Adopted: The 1-11–2 alert setting function classifies patients’ routine blood test data into Grade I, II, III, and IV. For each level, the system will immediately provide algorithm-based automatic grading and self-care management recommendations and advice based on evidence-based medical evidence.
1–9 Questionnaire	Expert 13: Can the questionnaire be set so that it can only be filled out within a specific time frame? This would prevent patients from completing the questionnaire too early or too late.	Accepted. Added a setting for the questionnaire completion time (1-9-5). Each questionnaire can be filled out within three days before or after the preset time.
1–11 Alert Handling	Expert 10: Regardless of the type of alert handled, it should be clearly recorded for later analysis.	Accepted. Any intervention triggered by an alert will be recorded in a custom patient follow-up record form.
2-1–3 Data Reports	Expert 1: Visualizing the main values of the complete blood count in the data reports would make it easier for users to view.	Accepted. The neutrophil count, white blood cell count, hemoglobin count, and platelet count uploaded by patients will be visualized using line charts, making it easier for users to view and understand.
2-2–2 Laboratory Records	Expert 12: The laboratory indicators in the complete blood count have different units. It would be best if the system automatically sets the units, so that patients know which value to enter.	Accepted. The system backend has automatically set the units for each laboratory indicator in the complete blood count.
2-2–3 Health Records	Expert 6: It is recommended to directly provide options for the general condition records here, allowing patients to choose for themselves and reducing their burden.	Accepted. Each item in the general condition records now directly provides options for patients to select.
Intelligent Reminders	Expert 4: Reminding patients three times for each complete blood count re-examination is too much; two times is sufficient. Expert 8: Two reminders are enough. You can remind at 8:00 p.m. the day before and at 4:00 a.m. on the day, so that patients have enough time to get their complete blood count re-examined.	Accepted. The number of reminders has been changed to two: patients will be reminded to re-examine their complete blood count at 20:00 the night before and at 14:00 on the day of the re-examination. If the patient has already completed the re-examination, the reminders will stop.
Team Division of Labor	Expert 9: The division of labor among team members is not detailed enough. It is recommended to further clarify the division of responsibilities and to use blinding as much as possible during evaluation to reduce bias.	Adopted. The division of responsibilities within the research team has been further refined, with each task assigned to a specific individual.
Compliance Issues	Expert 2: The issue of patient compliance should be more strictly managed.	Adopted. Patient activity on the platform will be monitored to track visit frequency. For patients who log in to the platform fewer than three times per month, researchers will contact them by phone to investigate the reasons for their limited participation. If poor compliance or other practical factors hindering compliance cannot be resolved, the patient will be withdrawn from the study.

#### 3.2.2 Second round

The second expert panel meeting focused on improving platform usability and interaction design. Experts provided specific recommendations to enhance operational efficiency, interaction modes, and visual clarity. In response, the research team further refined the platform structure. Detailed comparisons of expert feedback and subsequent revisions are summarized in **Table 5**. After two rounds of structured expert panel meetings, the research team refined the initial platform prototype and finalized the functional module framework and intelligent interaction engine for subsequent development. The final functional module framework and intelligent interaction engine are presented in **Supplementary Tables S6, S7** in **Supplementary Material 2**.

**Table 5:** Revision suggestions from the second expert meeting.

Function	Expert opinions	Modification plan
Due to forgetfulness, it is difficult to continuously and accurately upload routine blood test data during home care.	Expert 7: Increase the frequency of training sessions for patients and their families. Expert 2: Record an instructional video on how to use the platform to ensure that patients can continuously and accurately upload their complete blood count data while at home. Expert 6: The video can be uploaded to the department’s official WeChat account backend, and keywords can be set so that patients can access the instructional video by entering the relevant keywords.	Increase training frequency: In addition to instructing patients and their families on how to use the platform the day before chemotherapy, the teach-back method should be used on the day of chemotherapy to ensure they have mastered the operational essentials. At the same time, the recorded instructional video on platform usage should be uploaded to the department’s official WeChat account backend, with relevant keywords set, so that patients can access the video by entering the keywords while at home, helping them to continuously and accurately upload their complete blood count data.
Video follow-up issues	Expert 10: Considering that a large number of patients would require significant manpower and time, and that high server and network quality are needed, it is recommended to change the communication method to a combination of platform-based communication, telephone, and outpatient visits.	Cancel the video follow-up method; consultations will mainly be conducted through the healthcare communication module or by leaving messages at any time, which healthcare staff will check periodically.
Platform convenience	Expert 11: The system backend can be set to remember the password for the next login, reducing the burden on patients and optimizing the user experience.	Add a “remember password” function to the system backend, so that the mobile number and password are saved in the system. When logging in again after logging out, users can directly access the main page of the system without entering their account and password.
Font size issue	Expert 1: The average age of patients is generally between 45 and 55 years old. Please enlarge the font and interface on the platform to make it easier for users to read.	Enlarge the platform interface and font size.

### 3.3 Platform development

The BCI-MP platform was developed using web-based technologies and embedded in the department’s official WeChat public account. Users could register by scanning a QR code, after which information technology administrators assigned role-based access permissions to support standardized and secure user management. The platform was accessible across smartphones, tablets, and computers, thereby improving usability and accessibility.

**Figure 2** illustrates the patient-side interface of the BCI-MP platform. The patient-side system comprises five major functional modules: Health Education, Health Interaction, Message Center, Health Records, and User Center. The Health Education module provides disease-related education and chemotherapy-related guidance; the Health Interaction and Message Center modules support patient–clinician communication, notifications, and interactive support; and the Health Records module allows patients to record and review assessment scales, laboratory test results, health status, and medication use. The User Center supports personal information management, appointments, data reports, and password modification. Together, these modules are designed to support continuous self-management, communication, and follow-up during chemotherapy intervals.

**Figure 2:**
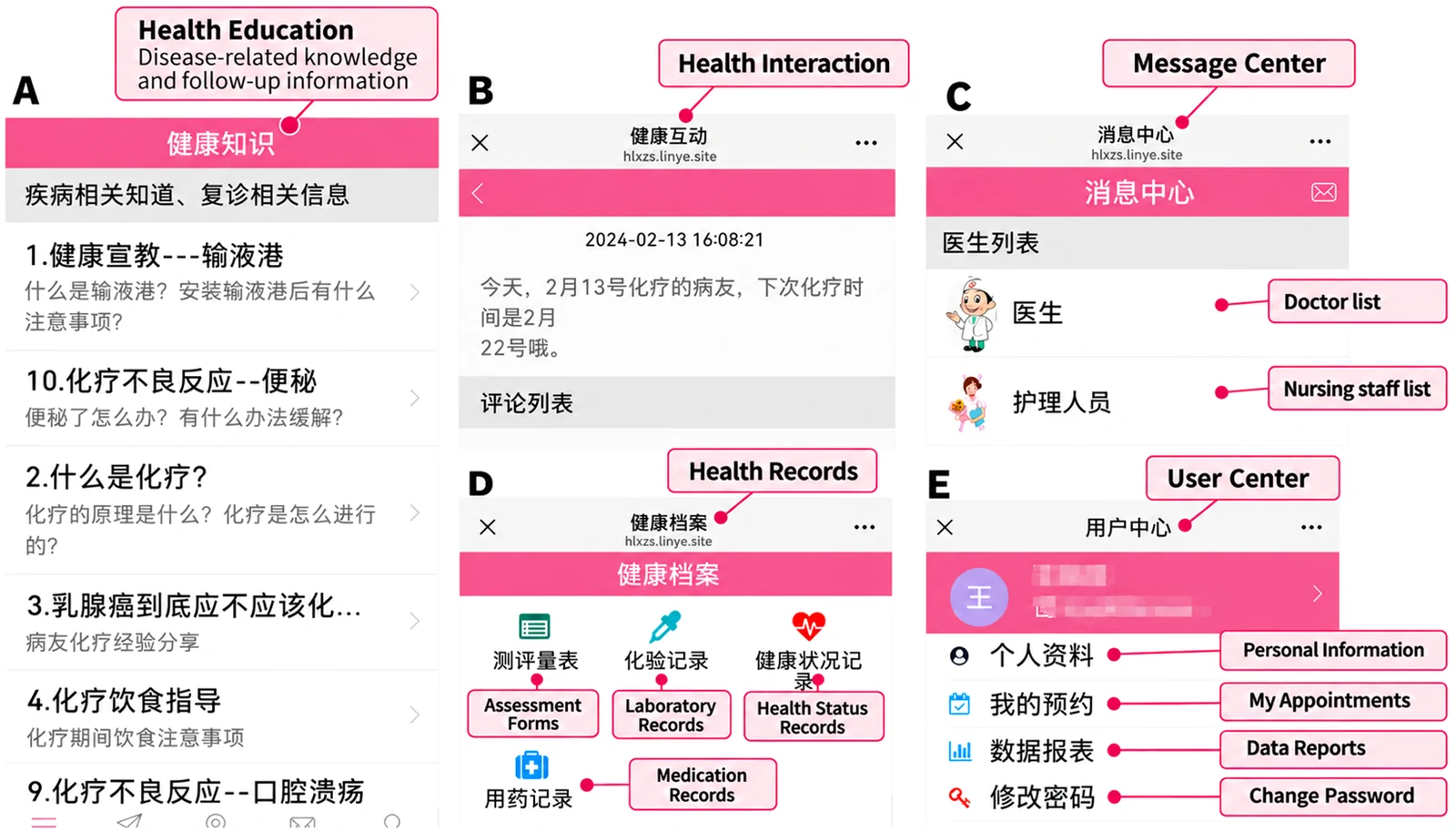
Patient-side interface. The patient-side interface **(A–E)** displays five major modules: Health Education, Health Interaction, Message Center, Health Records, and User Center. Screenshots retain the original Chinese-language interface; English annotations identify the key functional modules.

**Figure 3** presents the clinician-side interface and its core functions, including routine management, permission control, member administration, knowledge and appointment management, record maintenance, communication, intelligent alerts, health interaction, chemotherapy tracking, and SMS logging. The “Laboratory Records” module displays uploaded blood test results, whereas the “Health Management” module tracks daily indicators, including temperature, oral mucosa status, skin integrity, and bowel function. The “Myelosuppression Grading” feature applies World Health Organization criteria to automatically classify hematologic results and support rapid risk identification. The “Medication Records” module documents regular and chemotherapy-related medications separately to support medication review and adherence monitoring. The “Questionnaire Management” module enables clinicians to upload assessment tools with automated scoring, supporting real-time monitoring and individualized intervention.

**Figure 3:**
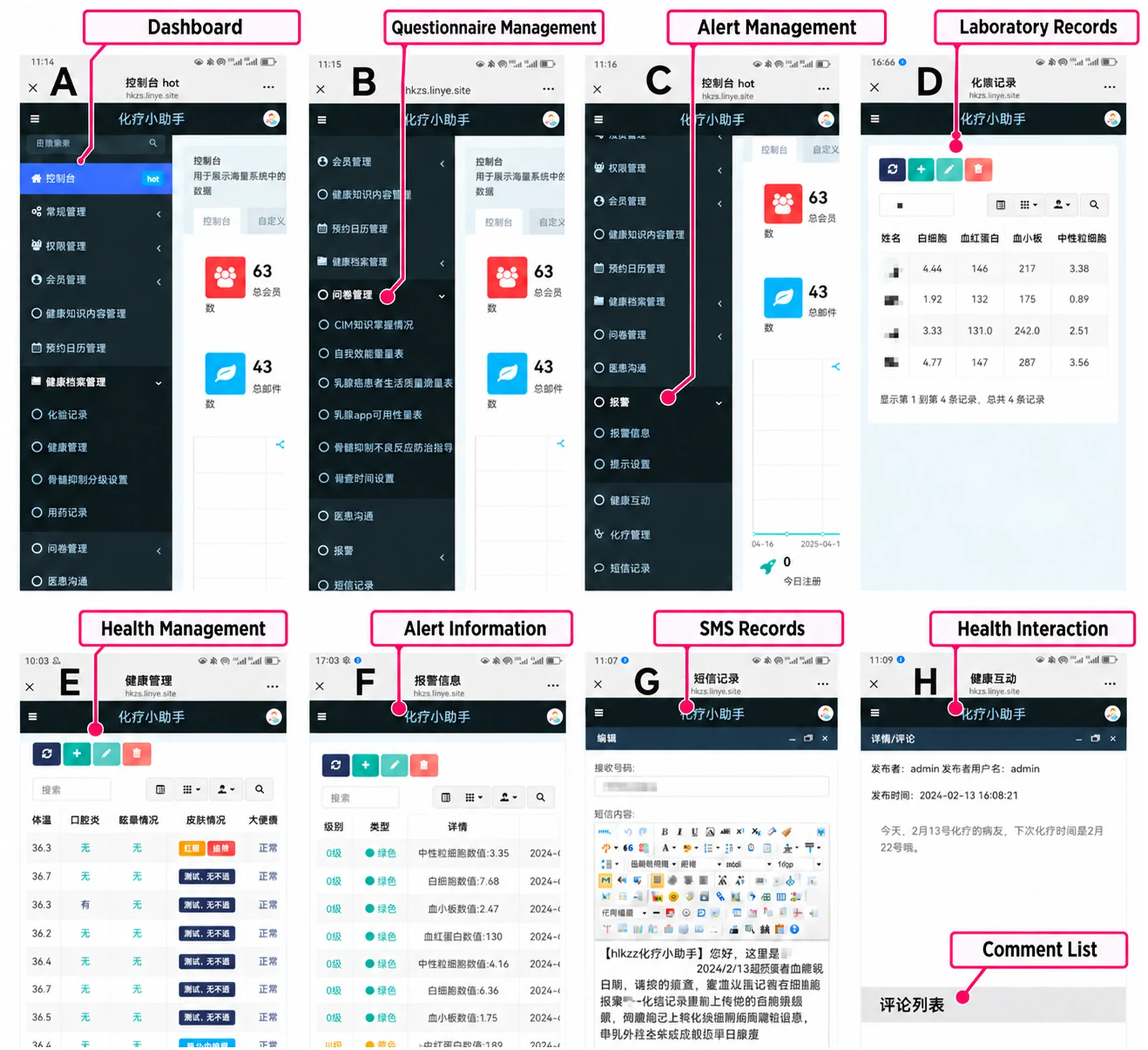
Clinician-side interface. The clinician-side interface **(A–C)** presents the main navigation interfaces for Dashboard, Questionnaire Management, and Alert Management. **(D–H)** illustrate the detailed functional interfaces for Laboratory Records, Health Management, Alert Information, SMS Records, and Health Interaction. Screenshots retain the original Chinese-language interface; English annotations identify the key functional modules.

The “Alert” module maps patient-uploaded complete blood count values to prespecified World Health Organization grading thresholds. Grade 0 represents normal hematologic findings and does not trigger a color-coded alert. Grades I, II, III, and IV trigger green, blue, yellow, and red alerts, respectively, with corresponding stratified management guidance. The “Chemotherapy Management” module records treatment cycles and schedules, enabling treatment tracking and adherence evaluation. The “Health Interaction” module allows clinicians to post updates or announcements that patients can view and comment on, supporting timely communication. Finally, the “SMS Records” module archives automated reminders, such as blood test and chemotherapy notifications, to enhance transparency, traceability, and continuity of care.

### 3.4 Pilot operation

#### 3.4.1 Participants

Eight patients with breast cancer who were initiating their first chemotherapy cycle were enrolled during the pilot phase. The mean age was 43.9 ± 10.9 years, and five participants were aged 30 to 49 years (5/8, 62.5%). Most participants were married (6/8, 75.0%), and two were divorced (2/8, 25.0%). Educational attainment included junior high school or below (5/8, 62.5%), high school or technical secondary school (2/8, 25.0%), and undergraduate education or higher (1/8, 12.5%). Six participants received adjuvant chemotherapy (6/8, 75.0%). Chemotherapy regimens included TAC (3/8, 37.5%), AC (2/8, 25.0%), TcbHP (1/8, 12.5%), ACT (1/8, 12.5%), and EC (1/8, 12.5%).

#### 3.4.2 Platform usability scores

The mean MAUQ domain scores were 3.72 ± 0.52 for Ease of Use and Satisfaction, 3.90 ± 0.38 for Information Architecture, and 3.27 ± 0.48 for Perceived Usefulness. Because lower MAUQ scores indicate higher usability, Perceived Usefulness had the lowest mean score among the three domains, whereas Information Architecture had the highest. The overall MAUQ score was 3.63 ± 0.32, suggesting moderate usability. The relatively higher score for Information Architecture suggests that information organization and interface layout may warrant further refinement.

#### 3.4.3 Platform usage

During the pilot phase, the platform sent 64 SMS reminders, recorded three sets of routine blood test results, and logged a mean of seven user sessions per participant (range, 3–14). Across the three uploaded complete blood count records, the platform generated 11 indicator-level alerts for abnormal hematologic findings, including five green (Grade I), two blue (Grade II), and four yellow (Grade III) alerts. No normal Grade 0 classifications were included in the alert count. Manual review confirmed that all 11 alert classifications were consistent with the prespecified World Health Organization grading thresholds for the corresponding hematologic values (11/11 concordance).

The platform maintained stable technical performance throughout the pilot phase. Data transmission, SMS delivery, and alert functions operated as expected, with no system crashes or data loss recorded. These findings support the preliminary technical feasibility of the platform for use in clinical chemotherapy-interval management.

## 4 Discussion

This study developed and preliminarily evaluated the BCI-MP platform, an mHealth tool embedded in an official WeChat public account, to support the management of chemotherapy-induced myelosuppression (CIM) during chemotherapy intervals in patients with breast cancer. In China, WeChat has been increasingly used for healthcare communication, patient education, and follow-up management (33), and embedding the platform within this widely used ecosystem may improve accessibility and implementation feasibility. The pilot evaluation provided preliminary insight into the platform’s usability and operational performance. The overall MAUQ score was 3.63 ± 0.32, indicating moderate usability and highlighting the need for further refinement, particularly in information architecture. In addition, the intelligent alert system generated 11 CIM-related alerts during the pilot phase and delivered corresponding stratified health guidance, indicating that these core functions were operational during the pilot.

The needs assessment revealed differences in perceived platform need and willingness to use the platform across user groups. Physicians reported the highest level of willingness, followed by nurses, whereas patients showed comparatively lower willingness to use the platform. This pattern may reflect differences in clinical roles, decision-making responsibilities, and perceived relevance to CIM management. Physicians, as key clinical decision-makers, require timely updates on patients’ conditions. Nurses, although central to patient education and follow-up care, generally have limited prescribing authority under China’s medical system (34, 35), which may influence how they prioritize decision-support functions. Patients’ willingness to use the platform may also be shaped by their awareness of CIM and individual health literacy (36). Moreover, actual engagement with the platform may be affected by demographic and contextual factors. For example, rural patients and some healthcare professionals may have concerns about usability, privacy, and additional workload (37–39). Importantly, 92.81% of patients had forgotten to recheck their blood tests, and 89.22% had experienced CIM. These findings are broadly consistent with the high incidence reported by Liu et al. (14) and highlight a substantial unmet need for structured CIM monitoring and follow-up support.

Compared with existing digital symptom management systems, the BCI-MP platform has several distinctive features for CIM monitoring and management. Many conventional alert systems generate warnings based on predefined indicators and focus primarily on early prevention (40). For example, Breen et al. (41) developed a tablet-based system for patients with hematologic malignancies that monitored adverse events but generated alerts only for febrile neutropenia. Similarly, Crafoord et al. (42) introduced an interactive application with symptom-triggered alerts; however, it did not include CIM-specific functionality. In contrast, the BCI-MP platform uses routine blood test data to support algorithm-based grading and alert generation while integrating automated reminders and individualized recommendations. This data-driven and interactive management model may help address limitations of conventional systems by linking objective laboratory data with remote monitoring and stratified guidance. However, its effects on patient safety, clinical responsiveness, and treatment adherence require further validation in larger controlled studies.

Given the challenge of sustaining engagement with mHealth applications among patients with cancer (43, 44), this study used a user-centered design approach that incorporated feedback from patients, clinicians, and multidisciplinary experts to enhance functional relevance and usability (45). Prior research suggests that co-design strategies can improve system usability and user satisfaction and may promote self-management behaviors (46–48). Although adherence was not formally evaluated in this phase, login frequency indicated that participants interacted with the platform. However, routine-care CBC results were not automatically synchronized with the BCI-MP and required patient-initiated uploading, and not all completed CBC results were uploaded during the pilot period. This explains why only three CBC records were captured and suggests that adherence to specific monitoring tasks may represent an important implementation challenge. Furthermore, embedding the platform within WeChat reduces the need for separate application installation and may lower access barriers. Built-in functions, such as clinician-patient messaging and visualized health data, may improve users’ comprehension and trust and support self-management (49–51).

Despite these preliminary strengths, several limitations should be considered. First, the pilot sample was small (n = 8), and the absence of a control group limited the generalizability of the findings and precluded conclusions regarding clinical effectiveness. Accordingly, the pilot should be interpreted as preliminary system testing intended to identify technical issues and assess initial usability rather than as a formal evaluation of clinical effectiveness. Second, although the platform incorporated interactive features to support communication and personalized care, heavy clinical workloads may limit timely responses from healthcare professionals outside regular working hours, thereby reducing system responsiveness. Third, embedding the BCI-MP within the WeChat ecosystem may introduce privacy and data-security concerns associated with reliance on a third-party platform. Although role-based access permissions were implemented, privacy and security were not formally evaluated and should be systematically assessed before broader clinical implementation. Future studies should therefore include larger controlled samples, longer follow-up, and mixed-methods evaluation to further assess usability, adherence, clinical effectiveness, implementation feasibility, and data privacy and security. Further optimization of clinician-side functions and systematic training to strengthen digital health competencies among healthcare professionals will also be important for improving the platform’s clinical applicability, scalability, and sustainability.

## 5 Conclusion

This study developed and preliminarily evaluated the BCI-MP platform, an mHealth intervention embedded in the WeChat ecosystem, to support chemotherapy-induced myelosuppression (CIM) management among patients with breast cancer undergoing chemotherapy. The platform enabled remote monitoring, automated alerts, and individualized health guidance and demonstrated moderate usability during pilot testing. Its intelligent alert system classified and flagged CIM-related abnormalities, supporting risk-stratified guidance and timely clinical follow-up. By integrating remote monitoring, automated alerts, and clinician-patient communication, the BCI-MP platform may help promote proactive health management, support treatment adherence, and streamline clinical follow-up processes. Furthermore, it may serve as a communication bridge among patients, clinicians, and specialized nurses, facilitating timely feedback and supporting a shift in nursing care from reactive responses toward proactive and preventive management. Future research should further examine its long-term clinical effectiveness, user adherence, implementation feasibility, and scalability to support broader integration into routine oncology practice.

## Data Availability

The raw data supporting the conclusions of this article will be made available by the authors, without undue reservation.
