# Supplementary material for: Designing and optimizing an intelligent health informatics management platform for patients undergoing chemotherapy for breast cancer: from needs assessment to pilot testing
Source: Front Oncol. 2026 Oct 22;16:1865670. doi: 10.3389/fonc.2026.1865670 (PMC13735732; doi:10.3389/fonc.2026.1865670)
Supplement: Supplementary file 1 [file DataSheet1.docx]

**Structure and Scoring Content of the Needs Assessment Questionnaire**

**A 1. Needs Assessment Questionnaire for the WeChat Platform for Bone Marrow Suppression during Breast Cancer Chemotherapy (Patient Version)**

Dear patients: Greetings!

We are currently conducting a project entitled "Design and Application of a Bone Marrow Suppression Management Platform during the Intermittent Period of Breast Cancer Chemotherapy." The aim is to provide you with a more personalized and real-time nursing platform, integrating reminders for routine blood re-examinations, detection and reporting, and early warning alerts for bone marrow suppression. This will help improve your knowledge of the disease, adherence to routine blood tests during chemotherapy intervals, and ultimately enhance your quality of life. In order to build an information platform that better meets patients' needs, is highly usable, and practical, we are conducting this needs assessment through the current questionnaire. Therefore, your opinions and suggestions are extremely important to us. This is an anonymous questionnaire and does not involve your personal privacy. We solemnly promise that your information will only be used for scientific research and will be kept strictly confidential. Please select the appropriate options based on your true thoughts and actual situation. We sincerely hope to receive your support and assistance. Thank you for your cooperation!

Instructions for Completion: Chemotherapy-induced bone marrow suppression refers to the reduction of white blood cells, hemoglobin, platelets, etc. in the blood. A decrease in white blood cells lowers the body's immunity and increases susceptibility to infection; a decrease in hemoglobin can lead to anemia, which may cause fatigue, weakness, and dizziness; a decrease in platelets may result in a tendency to bleed, such as easy bruising or uncontrolled bleeding. This questionnaire is a needs assessment for a mobile information platform for bone marrow suppression during daytime chemotherapy for breast cancer. From the second section onwards, the scoring standard adopts the five-point Likert scale: 1 = Not needed at all, 2 = Not much needed, 3 = Uncertain, 4 = Somewhat needed, 5 = Very much needed. Please tick "√" in the column that matches your opinion. Thank you for your cooperation!

A. Informed Consent Form

Do you agree to participate in this survey? □ Yes □ No ("Yes" proceeds to the next question; "No" ends the questionnaire)

B. General Patient Information Survey
1. Age: ______ years old
2. Gender: □ Male □ Female
3. Education level: □Primary school or below □Junior high school□High school/Technical secondary school □Junior college □Bachelor’s degree or above
4. How many months has it been since your last chemotherapy? □Don't remember
5. Have you ever forgotten the time for your routine blood test? □Yes □No
6. Have you ever experienced myelosuppression caused by chemotherapy? □Yes □No
7. Do you know that myelosuppression can be managed through the WeChat platform? □Yes □No
8. Have you ever used the WeChat platform to manage myelosuppression? □Yes □No
C. Willingness to use the platform
1. I need to use such a WeChat platform to manage myelosuppression

□Not needed at all	□Not really needed□Not sure □Somewhat needed□Very much needed

2. I am willing to use the WeChat platform to manage myelosuppression

□ Completely unwilling □ Somewhat unwilling □ Uncertain □ Somewhat willing □ Very willing□

D. Needs for Platform Functions and Content

| Serial Number | Item | Not Needed at All | Not Much Needed | Uncertain | Somewhat Needed | Highly Needed |
| --- | --- | --- | --- | --- | --- | --- |
| 1 | I need to access disease-related knowledge and coping methods at any time | 1 | 2 | 3 | 4 | 5 |
| 2 | I need to receive reminders before each routine blood test review | 1 | 2 | 3 | 4 | 5 |
| 3 | I need to know whether my routine blood test results are normal | 1 | 2 | 3 | 4 | 5 |
| 4 | I need the platform to provide me with targeted advice and guidance regarding abnormal blood routine results. | 1 | 2 | 3 | 4 | 5 |
| 5 | If my symptoms are severe, I need a quick way to contact the doctor for timely hospital return treatment. | 1 | 2 | 3 | 4 | 5 |
| 6 | I need to see the results of each blood routine test after the start of chemotherapy. | 1 | 2 | 3 | 4 | 5 |
| 7 | I need to keep in touch and communicate with fellow patients. | 1 | 2 | 3 | 4 | 5 |
| 8 | I need to keep in touch and communicate with medical staff. | 1 | 2 | 3 | 4 | 5 |

9. Please add any additional content you think should be included in the underlined area: __________________ ________________________________________________________________________________________________________________________________________________________________________________________________

**A 2. Survey on the Needs for a Bone Marrow Suppression Management Platform During the Chemotherapy Interval for Breast Cancer (Medical Staff Version)**

Dear Teachers, Greetings!

Thank you for taking the time out of your busy schedule to complete this questionnaire! I am Zhang Lin, a 2022 graduate student in Nursing at Zunyi Medical University. We are developing an intelligent management platform for bone marrow suppression during breast cancer chemotherapy, aiming to optimize the management process for bone marrow suppression in breast cancer chemotherapy patients in hospitals, promote the informatization of bone marrow suppression management, and provide a more personalized and real-time continuity of care platform for medical staff and patients. This platform will enable integrated, intelligent, and standardized management of CBC re-examination reminders, test result reporting, and bone marrow suppression early warning alerts, improve disease knowledge, enhance compliance with CBC re-examinations during chemotherapy intervals, reduce the incidence of bone marrow suppression, and improve both the work efficiency of medical staff and the quality of life of patients. In order to build an information platform that better meets the needs of medical management personnel, with strong usability and practicality, we are conducting this needs assessment through this questionnaire. Therefore, your opinions and suggestions are very important to us. This is an anonymous questionnaire and does not involve your personal privacy. We solemnly promise that your information will only be used for scientific research and will be kept strictly confidential. Please select the appropriate options based on your true thoughts and actual situation. We sincerely thank you for your support and cooperation!

Instructions for Completion: This questionnaire is a survey on the needs for a mobile information platform for bone marrow suppression management during day chemotherapy in breast cancer patients. Starting from the second section, the scoring standard adopts the five-point Likert scale: 1 = Not needed at all, 2 = Not really needed, 3 = Not sure, 4 = Somewhat needed, 5 = Very much needed. Please check “√” in the column that best reflects your opinion. Thank you for your cooperation!

A. Informed Consent Form

Do you agree to participate in this survey? □Yes □No (If yes, proceed to the next question; if no, the questionnaire ends)

B. General Information Survey for Medical Staff:
1. The hospital you work at is:
2. Your age is	years old
3. Your occupation: □Doctor □Nurse
4. Your educational background: □Technical secondary school □Junior college □Bachelor's degree □Master's degree □Doctoral degree
5. The number of years you have been working is	years
6. Have you ever encountered patients who forgot to recheck their complete blood count? □Yes □No
7. Have you ever managed patients who developed myelosuppression induced by chemotherapy? □Yes □No
8. Are you aware that myelosuppression-related management for patients can be conducted via the WeChat platform? □Yes □No
9. Have you ever used the WeChat platform to manage patients' myelosuppression? □Yes □No
C. Willingness to use the platform
1. I need to use the WeChat platform to manage patients' myelosuppression.

□Not needed at all □Not really needed □Not sure □Somewhat needed □Very much needed

2. I am willing to use the WeChat platform to manage patients' myelosuppression.

□Not willing at all □Not very willing □Not sure□□Somewhat willing □Very willing

D. Needs for the functions and content of the WeChat platfor

| Serial Number | Item | Not Needed at All | Not Much Needed | Uncertain | Somewhat Needed | Highly Needed |
| --- | --- | --- | --- | --- | --- | --- |
| 1 | I need to provide patients with disease-related knowledge and coping methods. |  |  |  |  |  |
| 2 | I need to remind patients to review their blood routine tests at appropriate times. |  |  |  |  |  |
| 3 | I need to automatically grade and alert for patients who have developed myelosuppression. |  |  |  |  |  |
| 4 | I need to promptly check the results of patients’ myelosuppression status. |  |  |  |  |  |
| 5 | I need to provide targeted guidance for patients with different grades of myelosuppression. |  |  |  |  |  |
| 6 | I need to promptly contact patients who have experienced severe bone marrow suppression adverse reactions and arrange for their return to the hospital for treatment. |  |  |  |  |  |
| 7 | I need the platform to visualize patients' longitudinal blood routine data. |  |  |  |  |  |
| 8 | I need to maintain contact and communication with patients. |  |  |  |  |  |

9. Please supplement with any additional content you think should be added in the space provided: ______________________________________________________________________________________________________________________________________________________________
