# Supplementary material for: Designing and optimizing an intelligent health informatics management platform for patients undergoing chemotherapy for breast cancer: from needs assessment to pilot testing
Source: Front Oncol. 2026 Oct 22;16:1865670. doi: 10.3389/fonc.2026.1865670 (PMC13735732; doi:10.3389/fonc.2026.1865670)
Supplement: Supplementary file 2 [file Table1.docx]

**Table S1: Initial Draft of Functional Modules**

| Functional Module | | Content Item | |
| --- | --- | --- | --- |
| 1 Medical Staff Side |  |  |  |
|  | 1-1 Console |  |  |
|  |  | 1-1-1 Total Number of Members |  |
|  |  | 1-1-2 Total Number of Administrators |  |
|  | 1-2 Personal Profile |  |  |
|  |  | 1-2-1 Avatar |  |
|  |  | 1-2-2 Username |  |
|  |  | 1-2-3 Mobile Number |  |
|  |  | 1-2-4 Email |  |
|  |  | 1-2-5 Nickname |  |
|  |  | 1-2-6 Edit |  |
|  |  | 1-2-7 Operation Log |  |
|  | 1-3 Permission Management |  |  |
|  |  | 1-3-1 Administrator Management |  |
|  |  |  | 1-3-1-1 Add Administrator |
|  |  |  | 1-3-1-2 Delete Administrator |
|  |  | 1-3-2 Administrator Log |  |
|  | 1-4 Membership Management |  |  |
|  |  | 1-4-1 Member Information |  |
|  |  |  | 1-4-1-1 Serial Number  1-4-1-2 Name  1-4-1-3 Gender  1-4-1-4 Age  1-4-1-5 Hospitalization Number  1-4-1-6 Phone Number  1-4-1-7 Profile Picture  1-4-1-8 Login Time  1-4-1-9 Registration Time  1-4-1-10 Marital Status  1-4-1-11 Educational Level  1-4-1-12 Monthly Household Income  1-4-1-13 Medical Expense Payment Method  1-4-1-14 Family Member's Phone Number  1-4-1-15 Disease Diagnosis  1-4-1-16 Disease Stage  1-4-1-17 Presence of Cancer Metastasis  1-4-1-18 Presence of Other Comorbidities  1-4-1-19 Chemotherapy Regimen  1-4-1-20 KPS Score |
|  |  | 1-4-2 View Laboratory Records | 1-4-2-1 White Blood Cell Count  1-4-2-2 Neutrophil Count |
|  |  |  | 1-4-2-3 Hemoglobin Level |
|  |  |  | 1-4-2-4 Platelet Count |
|  |  | 1-4-5 Login Records | 1-4-5-1 Login Time  1-4-5-2 Login Duration |
|  | 1-5 Health Knowledge |  |  |
|  |  | 1-5-1 Add  1-5-2 Edit  1-5-3 Delete  1-5-4 Refresh |  |
|  |  |  |  |
|  | 1-6 Appointment Calendar Management |  |  |
|  |  | 1-6-1 Add  1-6-2 Delete  1-6-3 Date Selection |  |
|  |  |  |  |
|  | 1-7 Health Record Management |  |  |
|  |  | 1-7-1 Laboratory Test Records |  |
|  |  |  | 1-7-1-1 Serial Number |
|  |  |  | 1-7-1-2 Username |
|  |  |  | 1-7-1-3 Name |
|  |  |  | 1-7-1-4 White Blood Cell Count |
|  |  |  | 1-7-1-5 Neutrophil Count |
|  |  |  | 1-7-1-6 Hemoglobin Value |
|  |  |  | 1-7-1-7 Platelet Count |
|  |  |  | 1-7-1-8 Time |
|  |  | 1-7-2 Health Management |  |
|  |  |  | 1-7-2-1 Serial Number |
|  |  |  | 1-7-2-2 Username |
|  |  |  | 1-7-2-3 Name |
|  |  |  | 1-7-2-4 Body Temperature |
|  |  |  | 1-7-2-5 Oral Mucositis |
|  |  |  | 1-7-2-6 Dizziness Status |
|  |  |  | 1-7-2-7 Skin Condition |
|  |  |  | 1-7-2-8 Stool Color |
|  |  |  | 1-7-2-9 Others |
|  |  |  | 1-7-2-10 Time |
|  |  | 1-7-3 Medication Record |  |
|  |  |  | 1-7-3-1 Serial Number |
|  |  |  | 1-7-3-2 Name |
|  |  |  | 1-7-3-3 Time |
|  | 1-8 Bone Marrow Suppression Grading Settings |  |  |
|  |  | 1-8-1 Grade 0 |  |
|  |  | 1-8-2 Grade I |  |
|  |  | 1-8-3 Grade II |  |
|  |  | 1-8-4 Grade III |  |
|  |  | 1-8-5 Grade IV |  |
|  | 1-9 Questionnaire Management |  |  |
|  |  | 1-9-1 Self-Efficacy Scale |  |
|  |  | 1-9-2 CIM Knowledge Mastery |  |
|  |  | 1-9-3 Mobile App Usability Scale |  |
|  |  | 1-9-4 Bone Marrow Suppression Adverse Reaction Prevention Satisfaction Scale |  |
|  | 1-10 Doctor-Patient Communication |  |  |
|  |  | 1-10-1 Medical Staff |  |
|  |  | 1-10-2 Patient |  |
|  | 1-11 Alert |  |  |
|  |  | 1-11-1 Alert Information |  |
|  |  |  | 1-11-1-1 Serial Number |
|  |  |  | 1-11-1-2 Name |
|  |  |  | 1-11-1-3 Contact Information |
|  |  |  | Level 1-11-1-4 |
|  |  |  | 1-11-1-5 Color |
|  |  |  | 1-11-1-6 Details |
|  |  |  | 1-11-1-7 Time |
|  |  | 1-11-2 Reminder Settings |  |
|  | 1-12 Health Interaction |  |  |
|  |  | 1-12-1 Serial Number |  |
|  |  | 1-12-2 Name |  |
|  |  | 1-12-3 Time |  |
|  |  | 1-12-4 Details |  |
|  | 1-13 Chemotherapy Management |  |  |
|  |  | 1-13-1 Serial Number |  |
|  |  | 1-13-2 Name |  |
|  |  | 1-13-3 Chemotherapy Time |  |
|  |  | 1-13-4 Remarks |  |
|  | 1-14 SMS Records |  |  |
|  |  | 1-14-1 Serial Number |  |
|  |  | 1-14-2 Name |  |
|  |  | 1-14-3 Sent Time |  |
|  |  | 1-14-4 Recipient Number |  |
|  |  | 1-14-5 Delete |  |
|  |  | 1-14-6 Editing |  |
| 2  Patient Side |  |  |  |
|  | 2-1 My |  |  |
|  |  | 2-1-1 Personal Information |  |
|  |  |  | 2-1-1-1 Name  2-1-1-2 Gender  2-1-1-3 Age  2-1-1-4 Hospitalization Number  2-1-1-5 Phone Number  2-1-1-6 Marital Status  2-1-1-7 Education Level  2-1-1-8 Monthly Household Income  2-1-1-9 Medical Expense Payment Method |
|  |  | 2-1-2 My Appointments |  |
|  |  |  | 2-1-2-1 Schedule Daytime Chemotherapy Appointment |
|  |  | 2-1-3 Data Reports |  |
|  |  |  | 2-1-3-1 Changes in White Blood Cell Count  2-1-3-2 Changes in Neutrophil Count  2-1-3-3 Changes in Hemoglobin Levels  2-1-3-4 Changes in Platelet Count  2-1-3-5 Self-Efficacy Score Status  2-1-3-6 Usability Scale Score |
|  |  | 2-1-4 Change Password |  |
|  |  |  | 2-1-4-1 Old Password  2-1-4-2 New Password  2-1-4-3 Confirm New Password |
|  |  | 2-1-5 Log Out |  |
|  |  |  | 2-1-5-1 Confirm Log Out |
|  | 2-2 Health Record |  |  |
|  |  | 2-2-1 Assessment Scales |  |
|  |  |  | 2-2-1-1 Self-Efficacy Scale  2-2-1-2 Knowledge Mastery Scale  2-2-1-3 APP Usability Scale  2-2-1-4 Satisfaction Scale for Prevention of Myelosuppression Adverse Reactions |
|  |  | 2-2-2 Laboratory Test Records |  |
|  |  |  | 2-2-2-1 Add  2-2-2-2 White Blood Cell Count  2-2-2-3 Neutrophil Count  2-2-2-4 Hemoglobin Value  2-2-2-5 Platelet Count |
|  |  | 2-2-3 Health Status Records |  |
|  |  |  | 2-2-3-1 Body Temperature  2-2-3-2 Stomatitis  2-2-3-3 Dizziness Status  2-2-3-4 Stool Color  2-2-3-5 Others |
|  |  | 2-2-4 Medication Records | 2-2-4-1 Medical Medication Records  2-2-4-2 Chemotherapy Medication Records |
|  | 2-3 Health Interaction |  |  |
|  |  | 2-3-1 Doctor-Patient Interaction |  |
|  |  |  | 2-3-1-1 Doctor-Patient Interaction  2-3-1-2 Patient Peer Interaction |
|  | 2-4 Message Center |  |  |
|  |  | 2-4-1 Chat Messages  2-4-2 System Messages |  |
|  |  |  | 2-4-1-1 Doctor  2-4-1-2 Nurse |
|  | 2-5 Health Knowledge |  |  |
|  |  | 2-5-1 Knowledge Related to Myelosuppression in Breast Cancer Chemotherapy |  |

**Table S2: Preliminary Design Draft of BCI-MP Intelligent Interaction Engine**

|  | Level | Content | |
| --- | --- | --- | --- |
|  | Function  Service Layer | ①Questionnaire: Edit the content and format of questionnaires, and distribute them. | |
|  |  | ②Resource Information: The researcher integrates clinical practice guidelines and expert consultations to convert offline health education content into text, images, audio, video, and other formats. The research team reviews and promptly revises the content as needed. After approval, the materials are uploaded to the platform for patients to study. Medical staff will also periodically upload information on disease knowledge, symptom management, coping skills, and social resources. At the same time, browsing traces (such as click counts, duration of browsing each module/post, etc.) and all patient monitoring data will be synchronously stored. | |
|  |  | ③Health Interaction: Provides a space for breast cancer day chemotherapy patients to raise questions, share experiences, and receive emotional support with other patients and/or medical staff, integrating knowledge acquisition and emotional communication. | |
|  |  | ④Chemotherapy Appointment List Display: Displays the chemotherapy appointment times for patients in the intervention group. The appointment list can be printed the day before chemotherapy. If a patient does not arrive on time for chemotherapy on the scheduled day, the patient is contacted promptly to inquire about the reason and resolve the issue in a timely manner to improve chemotherapy adherence and timeliness. | |
|  |  | ⑤Follow-up Tracking: Breast cancer chemotherapy patients can conduct case consultations within the system. Breast cancer specialist nurses provide timely one-on-one guidance and conduct follow-up visits on schedule (mainly through the chat interface on the platform and by phone), each session lasting 30–40 minutes. | |
|  | Intelligent Control Layer | ①Intelligent Reminder System: Based on the information provided by the patient, intelligent push reminders are set up. For example, patients are reminded to review their blood routine on the 3rd, 12th, and 20th days after chemotherapy, with reminders sent at 20:00 the night before, 09:00 and 14:00 on the day. If the patient has already completed the blood test, the reminders stop. Reminders for blood routine review and the next chemotherapy appointment are mainly sent via SMS. | |
|  |  | ②Intelligent Alarm System: The system immediately calculates the assessment results and intelligently identifies the symptom grading, displaying corresponding color-coded alerts. Grade I and II are low-risk warnings, indicating no abnormal symptoms or only mild symptoms; the alert colors are green and blue, respectively, and no action is required. Grade III is a medium-risk warning, indicating the patient has non-severe or non-life-threatening symptoms, but early intervention may prevent symptom progression or minimize harm; the alert color is yellow, and nurses are required to contact the patient within 4 hours of receiving the alert. Grade IV is a high-risk warning, indicating the patient has severe or even life-threatening symptoms; the alert color is red, and nurses must access the patient's visit information within 1 hour, contact the patient by phone as soon as possible to confirm the accuracy of the information, conduct a clinical assessment, and provide timely advice and support, such as immediately arranging an ambulance for the patient. | |
|  |  | ③Intelligent Analysis System: Any intervention triggered by an alert is recorded in the patient's medical record, serving the purpose of real-time remote monitoring and management. The system also intelligently analyzes patients' learning traces and the duration of browsing each module/post, intelligently identifies priority issues and patient preference information, and enables breast cancer chemotherapy patients to conduct self-health management. | |
|  |  | ④ Intelligent Appointment System: Based on the patient's first chemotherapy session, the system intelligently reminds the patient of the next chemotherapy date and prompts them to log into the platform to confirm the appointment. After the patient confirms the appointment, the system reminds them of pre-chemotherapy precautions, the chemotherapy process, and necessary items to bring. | |
|  |  | ⑤ Function Management: Responsible for components such as conversation history and topic management during a single round of interaction, integrating and connecting communication between medical staff and patients as well as among patients themselves, thereby enhancing social support for patients and achieving mutual support and improved patient compliance. | |
| Interaction Processing Layer | | In intelligent interactive management, the interface enables interaction between functional services and the intelligent control layer, including services such as backend management, control, and behavior analysis. Each user interface and its content can be operated independently without interference, enabling multidimensional interaction such as nurse-patient, patient-patient, human-computer, and information exchange. The main interaction methods include text, images, video, and telephone. |  |

**Table S3: Platform Usability Scale**

| Dimension | Item | Very Strongly Disagree | Strongly Disagree | Disagree | Neutral | Agree | Very Strongly Agree |
| --- | --- | --- | --- | --- | --- | --- | --- |
| Ease of Use and Satisfaction | 1. The application is easy to use. |  |  |  |  |  |  |
|  | 2. It is easy for me to learn how to use the application. |  |  |  |  |  |  |
|  | 3. I like the interface design of the application. |  |  |  |  |  |  |
|  | 4. The information in the application is well-organized, and I can easily find what I need. |  |  |  |  |  |  |
|  | 5. I feel comfortable using the application in public places. |  |  |  |  |  |  |
|  | 6. Using the application does not take too much of my time. |  |  |  |  |  |  |
|  | 7. I would use the application again. |  |  |  |  |  |  |
|  | 8. Overall, I am satisfied with the application. |  |  |  |  |  |  |
| System Information Arrangement | 1. I can easily and quickly correct any errors I make while using the application. |  |  |  |  |  |  |
|  | 2. The application provides healthcare services in a way that is acceptable to users. |  |  |  |  |  |  |
|  | 3. The application provides sufficient feedback and information to let me know my progress. |  |  |  |  |  |  |
|  | 4. The method and process of page switching are consistent throughout the application. |  |  |  |  |  |  |
|  | 5. The interface of the application allows me to use all its functions (such as inputting information, responding to reminders, and reading information). |  |  |  |  |  |  |
|  | 6. The application has all the features and capabilities I expect. |  |  |  |  |  |  |
| Usefulness | 1. The application is beneficial to my health. |  |  |  |  |  |  |
|  | 2. The application improves my access to healthcare services. |  |  |  |  |  |  |
|  | 3. The application helps me manage my health conditions more effectively. |  |  |  |  |  |  |
|  | 4. The application facilitates communication with my healthcare providers. |  |  |  |  |  |  |
|  | 5. The application provides me with more opportunities to communicate with my healthcare providers. |  |  |  |  |  |  |
|  | 6. I trust that my healthcare providers will receive any information I send via the application. |  |  |  |  |  |  |
|  | 7. I feel comfortable communicating with my healthcare providers through the application. |  |  |  |  |  |  |

**Table S4: General Information of Patients (N=306)**

| Item | Group | Frequency (n/%) |
| --- | --- | --- |

| Age (years) | ≤30 | 3(0.98) |
| --- | --- | --- |
|  | 31-40 | 33(10.78) |
|  | 41-50 | 109(35.62) |
|  | ≥50 | 161(52.61) |
| Gender | Female | 306(100.00) |
|  | Male | 0(0.00) |
| Educational Level | Primary school and below | 181(59.15) |
|  | Junior High School | 75(24.51) |
|  | Senior High School / Technical Secondary School | 26(8.50) |
|  | Junior College | 13(4.24) |
|  | Bachelor's Degree and Above | 11(3.59) |
| Months Since Last Chemotherapy | <3 | 91(29.74) |
|  | 3~5 | 113(36.93) |
|  | 6~9 | 74(24.18) |
|  | 10~12 | 28(9.15) |
| Whether the patient has ever forgotten the time for routine blood test follow-up | Yes | 284(92.81) |
|  | No | 22(7.19) |
| Have you ever experienced bone marrow suppression caused by chemotherapy? | Yes | 273(89.22) |
|  | No | 33(10.78) |
| Do you know that bone marrow suppression can be managed through the WeChat platform? | Yes | 86(28.10) |
|  | No | 220(71.90) |
| Have you ever used the WeChat platform to manage bone marrow suppression? | Yes | 18(5.88) |
|  | No | 288(94.12) |

**Table S5. General information of healthcare professionals in the needs survey (N = 164)**

| Item | Group | Frequency (n/%) |
| --- | --- | --- |
| Age (years) | ≤30 | 45(27.44) |
|  | 31–40 | 94(57.32) |
|  | 41–50 | 19(11.59) |
|  | ≥50 | 6(3.66) |
| Occupation | Doctor | 53(32.32) |
|  | Nurse | 111(67.68) |
| Educational Level | Technical Secondary School | 2(1.22) |
|  | Junior College | 27(16.46) |
|  | Bachelor's degree | 81(49.39) |
|  | Master's degree | 33(20.12) |
|  | Doctor | 21(12.80) |
| Years of Work Experience | <3 | 16(9.76) |
|  | 3~5 | 31(18.90) |
|  | 6~10 | 49(29.88) |
|  | >10 | 68(41.46) |
| Have you ever encountered patients who forgot to follow up on routine blood tests? | Yes | 137(83.54) |
|  | No | 27(16.46) |
| Have you ever managed patients who developed myelosuppression induced by chemotherapy? | Yes | 124(75.61) |
|  | No | 40(24.39) |
| Are you aware that bone marrow suppression management for patients can be conducted through the WeChat platform? | Yes | 101(61.59) |
|  | No | 63(38.41) |
| Have you ever used the WeChat platform to manage bone marrow suppression in patients? | Yes | 35(21.34) |
|  | No | 129(78.66) |

**Table S6. Final functional module framework of the BCI-MP platform**

| Functional Modules | | Content Items | |
| --- | --- | --- | --- |
| 1  Medical Staff Side | 1-1 Console |  |  |
|  |  | 1-1-1 Total Number of Members |  |
|  |  | 1-1-2 Total Number of Administrators |  |
|  | 1-2 Personal Profile |  |  |
|  |  | 1-2-1 Avatar |  |
|  |  | 1-2-2 Username |  |
|  |  | 1-2-3 Mobile Phone |  |
|  |  | 1-2-4 Email |  |
|  |  | 1-2-5 Nickname |  |
|  |  | 1-2-6 Modify |  |
|  |  | 1-2-7 Operation Log |  |
|  | 1-3 Permission Management |  |  |
|  |  | 1-3-1 Administrator Management |  |
|  |  |  | 1-3-1-1 Add Administrator |
|  |  |  | 1-3-1-2 Delete Administrator |
|  |  | 1-3-2 Administrator Log |  |
|  | 1-4 Member Management |  |  |
|  |  | 1-4-1 Member Information |  |
|  |  |  | 1-4-1-1 Serial Number  1-4-1-2 Name  1-4-1-3 Gender  1-4-1-4 Age  1-4-1-5 Hospitalization Number  1-4-1-6 Phone Number  1-4-1-7 Avatar  1-4-1-8 Login Time  1-4-1-9 Registration Time  1-4-1-10Marital Status  1-4-1-11 Education Level  1-4-1-12 Monthly Household Income  1-4-1-13 Medical Expense Payment Method  1-4-1-14 Family Member Phone Number  1-4-1-15 Disease Diagnosis  1-4-1-16 Disease Staging  1-4-1-17 Presence or Absence of Cancer Metastasis  1-4-1-18 Presence of Other Diseases  1-4-1-19 Chemotherapy Regimen  1-4-1-20 KPS Score  1-4-1-21 Edit |
|  |  | 1-4-2 View Laboratory Records |  |
|  |  |  | 1-4-2-1 Summary Trend Chart |
|  |  | 1-4-3Login Records |  |
|  |  |  | 1-4-3-1 Login Time  1-4-3-2 Login Duration |
|  | 1-5 Health Knowledge |  |  |
|  |  | 1-5-1 Add  1-5-2 Edit  1-5-3 Delete  1-5-4 Refresh |  |
|  |  |  |  |
|  | 1-6 Appointment Calendar Management |  |  |
|  |  | 1-6-1 Add  1-6-2 Delete  1-6-3 Date Selection |  |
|  | 1-7 Health Record Management |  |  |
|  |  | 1-7-1 Laboratory Test Records |  |
|  |  |  | 1-7-1-1 Serial Number |
|  |  |  | 1-7-1-2 Username |
|  |  |  | 1-7-1-3 Full Name |
|  |  |  | 1-7-1-4White Blood Cell |
|  |  |  | 1-7-1-5 Neutrophil |
|  |  |  | 1-7-1-6 Hemoglobin |
|  |  |  | 1-7-1-7 Platelet |
|  |  |  | 1-7-1-8 Time |
|  |  | 1-7-2 Health Management |  |
|  |  |  | 1-7-2-1 Serial Number |
|  |  |  | 1-7-2-2 Username |
|  |  |  | 1-7-2-3 Full Name |
|  |  |  | 1-7-2-4 Body Temperature |
|  |  |  | 1-7-2-5 Stomatitis |
|  |  |  | 1-7-2-6 Dizziness Condition |
|  |  |  | 1-7-2-7 Skin Condition |
|  |  |  | 1-7-2-8 Stool Color |
|  |  |  | 1-7-2-9 Others |
|  |  |  | 1-7-2-10 Time |
|  |  | 1-7-3 Medication Record |  |
|  |  | 1-7-4 |  |
|  |  |  | 1-7-4-1 Serial Number |
|  |  |  | 1-7-4-2 Name |
|  |  |  | 1-7-4-3 Time |
|  | 1-8 Bone Marrow Suppression Grading Configuration |  |  |
|  |  | 1-8-1 Grade 0 |  |
|  |  | 1-8-2 Grade I |  |
|  |  | 1-8-3 Grade II |  |
|  |  | 1-8-4 Grade III |  |
|  |  | 1-8-5 Grade IV |  |
|  | 1-9 Questionnaire Management |  |  |
|  |  | 1-9-1 Self-Efficacy Scale |  |
|  |  | 1-9-2 CIM Knowledge Mastery Status |  |
|  |  | 1-9-3 Mobile APP Usability Scale |  |
|  |  | 1-9-4 Bone Marrow Suppression Adverse Reaction Prevention and Treatment Satisfaction Scale |  |
|  |  | 1-9-5 Survey Time Settings |  |
|  | 1-10 Doctor-Patient Communication |  |  |
|  |  | 1-10-1 Medical Staff |  |
|  |  | 1-10-2 Patients |  |
|  | 1-11 Alarm |  |  |
|  |  | 1-11-1 Alarm Information |  |
|  |  |  | 1-11-1-1 Serial Number |
|  |  |  | 1-11-1-2 Name |
|  |  |  | 1-11-1-3 Contact Information |
|  |  |  | 1-11-1-4 Level |
|  |  |  | 1-11-1-5 Color |
|  |  |  | 1-11-1-6 Details |
|  |  |  | 1-11-1-7 Time |
|  |  | 1-11-2Reminder Settings |  |
|  | 1-12 Health Interaction |  |  |
|  |  | 1-12-1 Serial Number |  |
|  |  | 1-12-2 Name |  |
|  |  | 1-12-3 Time |  |
|  |  | 1-12-4 Details |  |
|  | 1-13 Chemotherapy Management |  |  |
|  |  | 1-13-1 Serial Number |  |
|  |  | 1-13-2 Name |  |
|  |  | 1-13-3 Chemotherapy Time |  |
|  |  | 1-13-4 Remarks |  |
|  | 1-14 SMS Records |  |  |
|  |  | 1-14-1 Serial Number |  |
|  |  | 1-14-2 Name |  |
|  |  | 1-14-3 Sending Time |  |
|  |  | 1-14-4 Acceptance Number |  |
|  |  | 1-14-5 Delete |  |
|  |  | 1-14-6 Edit |  |
| 2  PatientSide | 2-1 Mine |  |  |
|  |  | 2-1-1 Personal Information |  |
|  |  |  | 2-1-1-1 Name  2-1-1-2 Gender  2-1-1-3 Age  2-1-1-4 Hospitalization Number  2-1-1-5 Phone Number  2-1-1-6 Marital Status  2-1-1-7 Education Level  2-1-1-8 Monthly Household Income  2-1-1-9 Medical Expense Payment Method |
|  |  | 2-1-2 My Appointments |  |
|  |  |  | 2-1-2-1 Scheduled Daytime Chemotherapy Time |
|  |  | 2-1-3 Data Reports |  |
|  |  |  | 2-1-3-1 Summary Trend Chart  2-1-3-2 Self-Efficacy Score Trend Chart  2-1-3-3 Usability Scale Change Trend Chart |
|  |  | 2-1-4 Change Password | 2-1-4-1 Old Password  2-1-4-2 New Password  2-1-4-3 Confirm New Password |
|  |  | 2-1-5 Logout |  |
|  |  |  | 2-1-5-1 Log Out |
|  | 2-2 Health Records | 2-2-1 Assessment Scales |  |
|  |  |  | 2-2-1-1 Self-Efficacy Scale  2-2-1-2 Knowledge Mastery Scale  2-2-1-3 APP Usability Scale  2-2-1-4 Bone Marrow Suppression Adverse Reaction Prevention and Treatment Satisfaction Scale |
|  |  | 2-2-2 Laboratory Test Records |  |
|  |  |  | 2-2-2-1 White Blood Cell Count (× 10⁹/L)  2-2-2-2 Neutrophil Count (× 10⁹/L)  2-2-2-3 Hemoglobin Level (g/L)  2-2-2-4 Platelet Count (× 10⁹/L) |
|  |  | 2-2-3 Health Status Records |  |
|  |  |  | 2-2-3-1 Body Temperature  2-2-3-2 Stomatitis (Yes, No)  2-2-3-3 Dizziness Condition (Yes, No)  2-2-3-4 Skin condition (intact, no discomfort, red spots, erythema, itching, peeling of hands and feet)  2-2-3-5 Stool color (normal, black, dark red, fresh red)  2-2-3-6 Others (free text input) |
|  |  | 2-2-4 Medication records |  |
|  |  |  | 2-2-4-1 General medication records  2-2-4-2 Chemotherapy medication records |
|  | 2-3 Health interaction |  |  |
|  |  | 2-3-1 Doctor-patient interaction |  |
|  |  |  | 2-3-1-1 Doctor-patient interaction  2-3-1-2 Patient peer interaction |
|  | 2-4 Message Center |  |  |
|  |  | 2-4-1 Chat Messages  2-4-2 System Messages |  |
|  |  |  | 2-4-1-1 Doctor  2-4-1-2 Nurse |
|  | 2-5 Health Knowledge | 2-5-1 Knowledge Related to Bone Marrow Suppression in Breast Cancer Chemotherapy |  |

**Table S7. Final design of the intelligent interaction engine of the BCI-MP platform**

|  | Intelligent  Interaction  Engine | Content |
| --- | --- | --- |
|  | Function  Service Layer | ①Questionnaire: Edit the content and format of the questionnaire and distribute it, with a limited time for questionnaire completion. |
|  |  | ②Resource Information: The researcher personally combines clinical practice guidelines and expert consultations to convert offline health education content into text, images, audio, video, and other formats. The research team reviews these materials, makes timely modifications if necessary, and uploads the approved content to the WeChat platform for patient learning. Medical staff will periodically upload knowledge information such as disease knowledge, symptom management, coping skills, and social resources, with irregular updates to health knowledge. Meanwhile, browsing traces (such as click rates and the number of views of each health knowledge content module) and all patient test data will be synchronously stored. |
|  |  | ③Health Interaction: Provides a platform for breast cancer day chemotherapy patients to raise questions, share experiences, and offer emotional support with other patients and/or medical staff, integrating knowledge acquisition and emotional communication. |
|  |  | ④Chemotherapy Appointment List Display: Displays the chemotherapy appointment times for patients in the intervention group. The list can be printed one day before chemotherapy. If a patient does not arrive on time for chemotherapy on the scheduled day, the patient will be contacted promptly to inquire about the reason and resolve the issue in a timely manner, thereby improving patient adherence and timeliness of chemotherapy. |
|  |  | ⑤Follow-up Tracking: Breast cancer chemotherapy patients can conduct case consultations within the system, and breast specialist nurses provide timely one-on-one guidance (mainly through the platform’s chat interface and telephone). |
|  |  | ⑥Font and Size Settings: The platform interface and font size are enlarged. |
|  | Intelligent  Control Layer | ① Intelligent Reminder System: Based on the information provided by the patient, an intelligent push reminder function is set up, such as reminding the patient to undergo blood routine reexaminations on the 4th, 12th, and 20th days after chemotherapy. Reminders are sent at 20:00 the day before and 14:00 on the day of the reexamination. If the patient has already completed the blood routine test, the reminders will stop. The system sets reminders for blood routine reexaminations and the next chemotherapy appointment, mainly through WeChat and SMS notifications. |
|  |  | ② Intelligent Alarm System: Based on the World Health Organization (WHO) grading standards for acute and subacute toxic reactions to anticancer drugs as the criteria for bone marrow suppression, literature reviews are provided to establish bone marrow suppression grading and corresponding management measures. According to counts of white blood cells, neutrophils, hemoglobin, and platelets, the system classifies into Grade 0, I, II, III, and IV. It immediately calculates the assessment results and intelligently identifies symptom grading, displaying corresponding color-coded alerts. Grades I and II are low-risk warnings, indicating no abnormal symptoms or mild symptoms; the alert colors are green and blue respectively, requiring no intervention. Grade III is a medium-risk warning, indicating the patient has non-severe or non-life-threatening symptoms, but early intervention may prevent symptom progression or minimize damage. The alert color is yellow, requiring nurses to contact the patient within 4 hours of receiving the alert. Grade IV is a high-risk warning, indicating severe or life-threatening symptoms; the alert color is red, requiring nurses to access the patient’s medical information within 1 hour, contact the patient by phone as soon as possible to verify the accuracy of the information, conduct clinical assessment, and provide timely advice and support, such as immediately arranging an ambulance. Any intervention triggered by the alert is recorded in a custom patient follow-up record form. |
|  |  | ③ Intelligent Analysis System: Intelligently analyzes patients’ learning traces, the number of times various health knowledge items are viewed, login durations, etc., and intelligently identifies priority issues and patient preferences. Based on this intelligent analysis data, it provides data support for breast cancer chemotherapy patients to achieve self-health management. |
|  | | ④ Intelligent Appointment System: Based on the patient’s first chemotherapy date, the system intelligently reminds the patient of the next chemotherapy appointment and prompts the patient to enter the platform to confirm the appointment time. After the patient confirms the appointment, reminders are sent regarding pre-chemotherapy precautions, chemotherapy procedures, and necessary items to bring. |
|  |  | ⑤ Function Management: Sets features such as “Remember Password,” manages dialogue history and topic management during single-round interactions, and other Q&A functional components. It connects and integrates communication between doctors and patients as well as among patients themselves, enhancing patients’ social support to achieve mutual support and improve patient compliance. |
| Interaction  Processing Layer | | In the intelligent interactive management WeChat platform, the interface realizes the interaction between functional services and the intelligent control layer, including backend management and control, behavior analysis, and other services. Each user interface and its content can independently perform interactive operations without mutual interference, achieving multi-dimensional interaction modes such as nurse-patient interaction, human-computer interaction, and information exchange. The main interaction methods include text, images, and telephone. |
